# Synthetic Antiferromagnetic Gold Nanoparticles as Bimodal Contrast Agents in MRI and CT—An Experimental In Vitro and In Vivo Study

**DOI:** 10.3390/pharmaceutics13091494

**Published:** 2021-09-17

**Authors:** Antoine D’Hollander, Ruben Van Roosbroeck, Jesse Trekker, Tim Stakenborg, Tom Dresselaers, Greetje Vande Velde, Tom Struys, Ivo Lambrichts, Jeroen Lammertyn, Liesbet Lagae, Uwe Himmelreich

**Affiliations:** 1 Biomedical MRI Unit, Department of Imaging and Pathology, KU Leuven, O&N 1, Herestraat 49, 3000 Leuven, Belgium; antoinedhollander@hotmail.com (A.D.); jessetrekker@gmail.com (J.T.); tom.dresselaers@uzleuven.be (T.D.); Greetje.Vandevelde@kuleuven.be (G.V.V.); 2 Department of Life Science Technology, IMEC, Kapeldreef 75, 3001 Leuven, Belgium; ruben.vanroosbroeck@gmail.com (R.V.R.); Tim.Stakenborg@imec.be (T.S.); liesbet.lagae@imec.be (L.L.); 3 Division of Mechatronics, Department of Biosystems, Biostatistics and Sensors, KU Leuven, 3001 Leuven, Belgium; jeroen.lammertyn@kuleuven.be; 4 Lab of Histology, Biomedical Research Institute, Hasselt University, Agora Laan Gebouw C, 3590 Diepenbeek, Belgium; tom.struys@sprofit.com (T.S.); ivo.lambrichts@uhasselt.be (I.L.); 5 Department of Physics, Faculty of Sciences, Laboratory of Soft Matter and Biophysics, KU Leuven, Celestijnenlaan 200D, 3001 Leuven, Belgium

**Keywords:** magnetic resonance imaging, MRI, computed tomography, CT, antiferromagnetic nanoparticles, gold nanoparticles, top down synthesis, bimodal contrast agent

## Abstract

The use of multimodal contrast agents can potentially overcome the intrinsic limitations of individual imaging methods. We have validated synthetic antiferromagnetic nanoparticles (SAF-NPs) as bimodal contrast agents for in vitro cell labeling and in vivo cell tracking using magnetic resonance imaging (MRI) and computed tomography (CT). SAF-NP-labeled cells showed high contrast in MRI phantom studies (r_2_* = 712 s^−1^ mM^−1^), while pelleted cells showed clear contrast enhancement in CT. After intravenous SAF-NP injection, nanoparticles accumulated in the liver and spleen, as visualized in vivo by significant MRI contrast enhancement. Intravenous injection of SAF-NP-labeled cells resulted in cell accumulation in the lungs, which was clearly detectable by using CT but not by using MRI. SAF-NPs proved to be very efficient cell labeling agents for complementary MRI- and CT-based cell tracking. Bimodal monitoring of SAF-NP labeled cells is in particular of interest for applications where the applied imaging methods are not able to visualize the particles and/or cells in all organs.

## 1. Introduction

Nanoparticles have proven their value as agents in life science applications such as cancer theranostics or therapeutic cell imaging [1,2,3,4]. Cell-based therapy makes use of therapeutic cells such as stem cells, dendritic cells or T-cells for the treatment of diseases ranging from cancer to neurological and cardiac diseases [4,5,6,7]. Crucial is the ability to follow the distribution and migration of these cells after injection to evaluate the efficacy of these therapies in a preclinical context. By either using direct, e.g., nanoparticles, or indirect labeling using genetically modified cells, several imaging techniques have been introduced to follow therapeutic cells [3,4,5,6,7,8,9,10,11,12,13,14]. The most prominent of these imaging techniques are magnetic resonance imaging (MRI), positron emission tomography (PET), X-ray based computed tomography (CT) and optical imaging. All of these have their specific advantages and disadvantages. PET and optical imaging are highly sensitive but PET suffers from short-lived tracers, whereas optical imaging is often limited by its low penetration depth. MRI and CT both provide anatomical data with high spatial resolution. However, CT is limited due to its poor soft-tissue contrast. MRI lacks performance in regions of low proton density, such as the lungs and bones. Recently, major interest has been shown in hybrid nanostructures that integrate different material components to be used for multiple imaging techniques simultaneously, thereby overcoming the limitations of single modality nanoparticles and imaging. This has already led to the use of hybrid nanoparticles in dual-mode and multi-modal molecular imaging techniques, such as MRI and fluorescence imaging [15,16], MRI and PET [17,18,19], MRI and photoacoustic imaging [20], CT and photoacoustic imaging [21], MRI and CT [22] and more. Comparing the latter two techniques, on the one hand CT is advantageous with regard to its higher resolution and contrast generated between calcified tissue, air and soft tissue, but it exhibits poor soft-tissue contrast and relatively low sensitivity to contrast agents. On the other hand, MRI displays a higher sensitivity for contrast agents and excellent soft-tissue contrast, but the negative, hypointense contrast generated by iron oxide-based nanoparticles is difficult to quantify and can be confounded with other hypointense areas, resulting from air, bleeding and calcification [14]. Bimodal MRI/CT imaging can thus overcome the limitations of each individual technique in specific organ systems, which is in particular important for studying the whole-body distribution of engrafted cells such as stem cells, immune cells or pancreatic islets. Moreover, hybrid nanoparticles that target these applications have the potential for easy translation to the clinic, as CT and MRI are frequently used tools in clinical diagnosis. Both for MRI and CT, nanoparticles have been used and optimized to increase the contrast and sensitivity. Magnetic nanoparticles have been extensively explored as negative contrast agents in T_2_*-weighted MRI [9,14,23,24]. For CT, electron-dense particles have been proven most beneficial [25,26]. Especially gold nanoparticles have been manifested as better CT contrast enhancers compared to clinical agents such as iodine-based compounds [27,28,29]. Combining gold and magnetic materials in one particle can result in bimodal nanoparticles that may be used as contrast agents in both MRI and CT. Such hybrid nanoparticles have already been synthesized using different techniques, resulting in hybrid nanostructures, including core-shell NPs [30,31,32] and dumbbell-like structures [33,34]. These particles were mostly bottom-up synthesized, resulting in complex synthesis procedures and polydisperse nanoparticle suspensions [35]. Moreover, the successive plating of gold layers around the magnetic core can result in a vast reduction of the T_2_*-contrast enhancement in MRI [36]. As an alternative, we propose top–down synthesized, gold synthetic antiferromagnetic nanoparticles (SAF-NPs) as bimodal MRI/CT contrast agents that do not suffer from these limitations. In previous work, we have already demonstrated the performance of SAF-NPs as T_2_* MRI contrast agents with high r_2_*-relaxivities of up to 355 s^−1^ mM^−1^ [37]. The results for SAF-NPs excel any other report on the T_2_* relaxivity of gold–magnetic hybrid nanoparticles. The easy synthesis method makes the introduction of gold as capping layer possible to also induce contrast in CT. Here, we present the in vitro and in vivo validation of SAF-NPs as contrast agents for tracking engrafted cells by using MRI and CT. We address their potential for in vivo imaging in two different scenarios, using intravenous injection of nanoparticles and tracking of cells pre-labeled with the nanoparticles.

## 2. Materials and Methods

### 2.1. Nanoparticle Synthesis and Functionalization

SAF-NPs with a layered structure of [Au (10 nm)/Ni_80_Fe_20_ (10 nm)/Au (2.5 nm)/Ni_80_Fe_20_ (10 nm)/Au (10 nm)] and a diameter of 90 nm and 222 nm were synthesized and functionalized as described previously [37]. As illustrated previously [37], starting from a wafer, the different materials (resist, magnetic stack) are added stepwise. Subsequently, polystyrene beads are drop casted as an etch mask for an ion milling step. The polystyrene beads are then removed using oxygen plasma treatment.

### 2.2. Cell Culture

SKOV3 cells (ATCC^®^HTB77, ATCC, Manassas, VA, USA) were cultured in RPMI 1640 medium supplemented with 10% Fetal Calf Serum (FCS), 50 units/L penicillin and 50 µg/mL streptomycin and 1 × 10^−5^ mol L-glutamine. Cells were incubated at 37 °C in a 5% CO_2_ environment. All cell culture reagents were obtained from Life Technologies (Ghent, Belgium). The SKOV3 cells were transduced with a lentiviral vector (LV-CMV-eGFP-T2A-fLuc) to stably express eGFP and firefly luciferase [38].

### 2.3. Confirmation of Cell Labeling In Vitro

For uptake confirmation, 500,000 cells per well were seeded in a 6 well plate. SAF-NPs (12 µg of particles, based on the Ni_80_Fe_20_ concentration, in 1 mL) were added after the cells attached to the substrate (typically after 24 h) and incubation continued for an additional 24 h. Two different sizes of nanoparticles were used, namely, 89.8 ± 18.6 (90) nm and 222.3 ± 9.1 (222) nm SAF-NPs. Next, cells were washed with phosphate buffer saline (PBS) and again incubated overnight with fresh, SAF-NP-free medium. After trypsinization, 100,000 cells were acid-digested with aqua regia (HCl/HNO_3_ with a ratio of 3:1) and diluted with deionized water to a volume of 10 mL for inductively coupled plasma—optical emission spectroscopy (ICP-OES, 3300 DV, Perkin-Elmer, Waltham, MA, USA). Reference standards were prepared by dissolving Au, Ni and Fe standards (Merck, Overijse, Belgium) to final concentrations between 0.1 and 5 ppm.

### 2.4. Transmission Electron Microscopy (TEM)

For TEM analysis, labeled cells were seeded on plastic Nunc™ Thermanox™ coverslips (Laborimpex, Brussels, Belgium) and fixed with 2% glutaraldehyde in 0.05 M sodium cacodylate buffer (pH = 7.3) (Sigma Chemical Co, St. Louis, MO, USA) at 4 °C. Following fixation, the samples were washed twice for 5 min with 0.05 M sodium cacodylate (pH = 7.3) and 0.15 M saccharose (Sigma Chemical Co, St. Louis, MO, USA) at 4 °C. Postfixation was achieved by treating the samples with 2% osmiumtetroxide (Aurion, Wageningen, The Netherlands) in 0.05 M sodium cacodylate buffer (pH = 7.3) for 1 h at 4 °C. The samples were dehydrated by exposing them to increasing concentrations of acetone (Honeywell, Raunheim, Germany). Next, the samples were impregnated overnight in a 1:1 mixture of acetone and araldite epoxy resin (Aurion) at room temperature. In a following step, the samples were embedded in araldite epoxy resin at 60 °C. After applying the pop-off method, the embedded samples were cut in sections of 40–60 nm, using a Leica EM UC6 microtome (Leica, Wetzlar, Germany). Sections were then transferred to 50 mm mesh copper grids (Aurion) coated with 0.7% formvar (Sigma Chemical Co). The samples were automatically stained using a Leica EM AC20 (Leica) with 0.5% uranyl acetate and a stabilized solution of lead citrate (both from Laurylab, Brindas, France). TEM was performed with an EM208 S electron microscope (Philips, Best, The Netherlands) operated at 80 kV and digital processing of the images was done with iTEM-FEI software (Olympus SIS, Aartselaar, Belgium).

### 2.5. Animal Model

Hsd:Athymic Nude-Foxn1nu6 mice were used (6 weeks, female, Harlan, Horst, The Netherlands) during these experiments. All animal experiments were approved by the animal ethics committee of KU Leuven (project number: P259/2015, date: 15 December 2015, date amendment: 24 July 2019). All procedures were performed according to the national and European regulations. Animals were housed in individually ventilated cages with free access to food and water. During all imaging experiments and cell injections, the animals were anesthetized with 1.5% isoflurane in pure oxygen while monitoring the respiration rate and maintaining the body temperature at 37 °C ± 0.5 °C. To determine the performance of the SAF-NPs as bimodal contrast agents in MRI and CT, both phospholipid-coated SAF-NPs (100 µL of 1 × 10^8^ NPs/mL) or SAF-NP-labeled SKOV3 cells (500,000 cells in 200 µL PBS) were intravenously injected into the tail vein of mice. All experiments were performed in triplicates.

### 2.6. In Vivo Bioluminescence Imaging (BLI)

Twenty-four hours after intravenous injection with 500,000 unlabeled and SAF-NP-labeled bioluminescent SKOV3 cells, mice were imaged with BLI upon intravenous injection of D-luciferin (126 mg/kg, Promega, Madison, WI, USA) dissolved in PBS (15 mg/kg). Mice were placed in an in vivo IVIS 100 imaging system in the prone position (Perkin Elmer, Waltham, MA, USA) using the following settings: 1 s exposure time and an FOV of 10 cm, binning of 4 and f/stop of 8. For the quantification of the fLuc reporter gene activity in vivo, the data were analyzed using Living Image software (v. 2.50.1) and presented as the photon flux (p/s) from a 2 cm^2^ circular ROI covering the lungs.

### 2.7. Computed Tomography (CT)

For in vitro CT contrast determination, a phantom was produced consisting of different amounts of labeled cells (5000 to 500,000) in PBS, pelleted in 15 mL tubes and covered with 1.5% agar. CT images were acquired using an in vivo microCT scanner (Skyscan 1076, Bruker microCT, Kontich, Belgium) with the following settings: 50 kV X-ray source, 200 µA source current, 0.5 mm Al filter, 120 ms exposure time and 0.7° rotation step, to result in images with a 35 µm isotropic resolution. The resulted tomograms were reconstructed using NRecon software (Version 1.6.1.3., Skyscan) with a smoothing of ‘3’, beam-hardening correction of ‘8%’ and the minimum and maximum for image conversion were 0.003 and 0.015. These reconstructed images were visualized using Dataviewer (Skyscan) and the grey density calculated by drawing a ROI in the lungs using CTan (Skyscan).

### 2.8. Magnetic Resonance Imaging (MRI)

To prepare the cells for in vitro MRI, a phantom was made as previously described [39]. A cylindrical holder (diameter 7 cm) containing 1.5% agar gel (Sigma Aldrich, St. Louis, MO, USA) was filled with imprints from microcentrifuge tubes was filled with different amounts of unlabeled and SAF-NP-labeled cells (5000 to 500,000) suspended in 2% liquid agar in 100 µL PBS. As control, the same amount of unlabeled cells was added. MR images of the phantom were acquired with a Bruker Biospec 9.4 T small animal MR scanner (Bruker Biospin, Ettlingen, Germany; horizontal bore, 20 cm) equipped with actively shielded gradients (600 mT.m^−1^). A quadrature radio-frequency transmit/receive resonator (inner diameter 7 cm, Bruker Biospin) was used for data acquisition. Measurements of the T_2_* relaxation times were performed using a multigradient echo pulse sequence with 8 echo time (TE) increments (repetition time (TR) = 1500 ms, first TE = 4.44 ms with increments of 6.75 ms, 400 × 400 matrix, 187.5 × 187.5 μm in plane resolution, 0.35 mm slice thickness, 12 slices). ImageJ (NIH, Bethesda, MD, USA) was used for further image processing. Signal intensities over echo-times were determined as the mean values of one slice of a homogeneous section of the cell loaded areas in the agar phantoms. The T_2_* relaxation times of the respective regions of interest were calculated from the best-fit least square first order exponential decay line with variable offset of the measured values. For in vivo MRI, mice were scanned with the same 9.4 T Bruker Biospec small animal MR scanner. Animals were scanned 24 h after injection of SAF-NPs or labeled cells. The in vivo MRI protocol used for imaging the torso of the mice consisted of a 2D T_2_* weighted fast low-angle shot (FLASH) and a multi-slice-multi-echo (MSME) sequence. The FLASH sequence (TE = 2.3 ms, TR = 203 ms, 6 slices with a thickness of 1 mm each and an in-plane resolution of 117 µm^2^) was used to determine the decrease in the signal intensity (SI) post injection. T_2_ values (maps) were determined from the MSME experiments and were used for a semi-quantitative analysis Parameters for the MSME sequences were a TR of at least 3000 ms, echo spacing of 7 ms and an 234 μm^2^ in-plane resolution with six slices of 1 mm thickness each.

### 2.9. Ex Vivo Dark-Field Microscopy

After imaging, the mice were sacrificed and perfused with 4% paraformaldehyde (PFA) and the organs were dissected (i.e., liver, heart, kidney, lung and spleen). These organs were stored in PBS containing 0.1% sodium azide (Sigma Aldrich) at 4 °C. After paraffin embedding, the tissues were sliced in 7 μm sections and deparaffinized before imaging using dark-field microscopy.

### 2.10. Ex Vivo TEM

As with dark-field microscopy, the animals were sacrificed and perfused with 4% PFA for ex vivo TEM imaging. Small pieces of the tissue samples were fixed overnight in 2% glutaraldehyde in 0.05 M sodium cacodylate buffer (pH 7.3; Sigma Aldrich,) at 4 °C, and post-fixed in 2% OsO_4_ in 0.05 M sodium cacodylate buffer (pH 7.3; Sigma Aldrich) for 1 h. Samples were then dehydrated in graded concentrations of acetone and embedded in epoxy resin (Araldite). Semi-thin slices (500 nm) were cut, stained with toluidine blue (Sigma Aldrich) and used for determining regions of interest. From the selected tissue blocks, ultra-thin sections (60 nm) were cut and mounted on 0.7% formvar coated grids, contrasted with uranyl acetate, followed by lead citrate, and examined in a Philips EM 208 transmission electron microscope operated at 80 kV. Images were taken with a MORADA 10/12 camera (Olympus).

### 2.11. Statistical Analysis

Differences between variables between the control and SAF-NPs batches were analyzed using GraphPad Prism software (GraphPad Software Inc., La Jolla, CA, USA), where an unpaired t-test was used with equal variance and the level of significance was set at 0.05. The levels of significance are shown in the figures as *: *p* < 0.05; **: *p* < 0.01; and ***: *p* < 0.001.

## 3. Results

### 3.1. Nanoparticles and Cell Labeling

**Figure 1:**
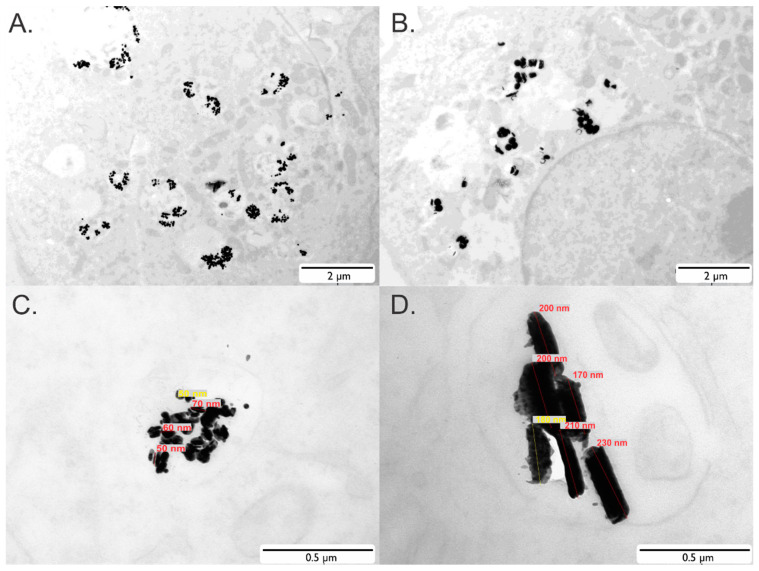
In vitro cell uptake of SAF-NPs. (**A**,**B**) TEM images of a single SAF-NP-labeled cell after overnight incubation with 90 nm (**A**) and 222 nm (**B**) SAF-NPs, revealing particle entrapment in small vesicles inside the cell. (**C**,**D**) Zoomed-in view of the SAF-NPs in labeled cells, indicating the size and disc-shape of the nanoparticles.

We have evaluated the in vitro and in vivo performance of SAF-NPs as bimodal MRI and CT contrast agents. Synthesis and characterization of the SAF-NPs resulted in identical parameters as described before [37], confirming the high reproducibility of particles with different diameters (90 nm and 222 nm). Using ICP-OES, a ratio between the Ni_80_Fe_20_ and the Au content (mass (Au)/mass (Ni_80_Fe_20_) was 4.4 for the 90 nm SAF-NPs and 3.3 for the 222 nm SAF-NPs, respectively. As shown by transmission electron microscopy (TEM), internalization and uptake of the SAF-NPs was clearly demonstrated for by SKOV3 cells for SAF-NPs with a diameter of 90 nm (Figure 1A,C) and 222 nm (Figure 1B,D). From these images, it is apparent that the SAF-PL-NPs were internalized by the cells and clustered within endosomal compartments of the cytoplasm. The number of internalized particles is larger for SKOV3 cells labeled with 90 nm SAF-NPs compared to 222nm SAF-NPs. Using inductively coupled plasma—optical emission spectrometry (ICP-OES), the Au and Ni_80_Fe_20_ concentration for 90 nm SAF-NP-labeled cells were determined to be 18.6 ± 0.1 pg/cell and 4.2 ± 0.1 pg/cell, respectively. For the 222 nm SAF-NP-labeled cells, the Au concentration was 24.7 ± 0.1 pg/cell and 7.9 ± 0.2 pg/cell for NiFe. This corresponds to approximately 1300 particles/cell for 90 nm SAF-NP-labeled cells and 350 particles/cell for 222 nm SAF-NP-labeled cells, respectively. Relative to the original amount of added particles, 20–30% of all particles were internalized using either the 90 nm or 222 nm SAF-NPs. For all in vitro and in vivo MRI and CT experiments with SAF-NP-labeled cells, similar amounts of nanoparticle labeling were achieved. Efficient cell uptake of both 90 nm and 222 nm NPs was thus confirmed and no clear difference in the final NP content could be observed between the different sizes comparing the Au and Ni_80_Fe_20_ amount/cell.

### 3.2. In Vitro Imaging Experiments

#### 3.2.1. Magnetic Resonance Imaging (MRI)

**Figure 2:**
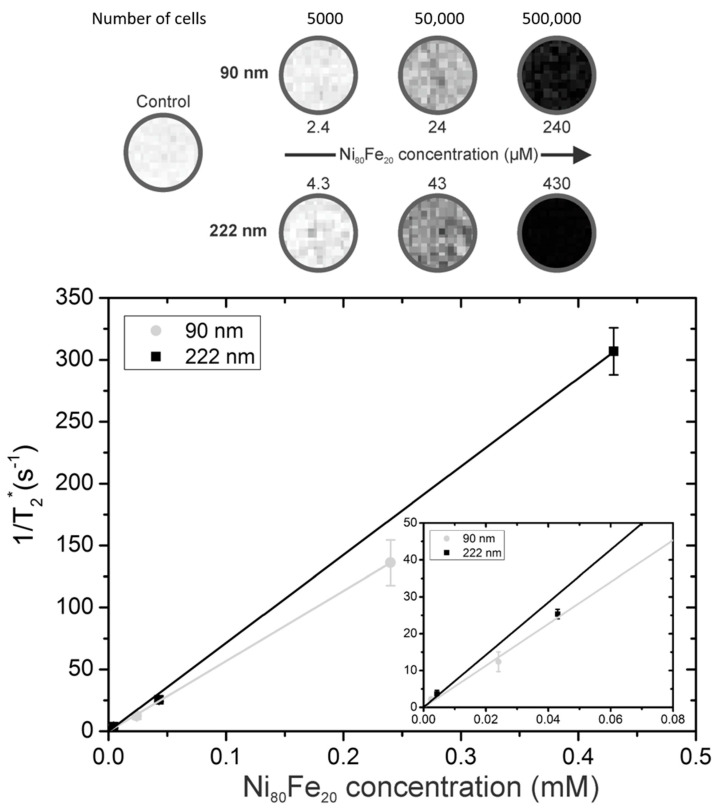
MRI of the SAF-NP labeled cells. (top) T2*-weighted MR images of the homogeneously suspended SAF-NP-labeled SKOV 3 cells in an agar phantom at an echo time of 38 ms for the given cell number and Ni_80_Fe_20_ concentration. The control sample contained 500,000 unlabeled cells. (bottom) Plot of the resulting 1/T2* values as a function of the intracellular Ni_80_Fe_20_ concentration. The inset is a magnification at lower Ni_80_Fe_20_ concentrations.

To assess the transverse relaxivity (r_2_*) of the SAF-NP-labeled cells, cells were homogeneously suspended in a phantom at different cell densities. The T_2_*-weighted MR images are shown in Figure 2. Starting from a control sample (500,000 unlabeled cells) to 5000, 50,000 and 500,000 labeled cells, the negative contrast gradually increased corresponding to the increasing Ni_80_Fe_20_ concentration (2.4 to 240 µM and 4.3 to 430 µM for the 90 nm and 222 nm SAF-NP-labeled cells, respectively). The r_2_*-relaxivities were determined by plotting the resulting unlabeled cell containing agar subtracted transverse relaxation rates (1/T_2_*) in function of the total Ni_80_Fe_20_ concentration (Figure 2). This resulted in relaxivities of r_2_* = 566 ± 18 s^−1^ mM^−1^ for the 90 nm SAF-NP-labeled cells and 712 ± 19 s^−1^ mM^−1^ for the 222 nm SAF-NP-labeled cells. For the 222 nm SAF-NPs, similar results were thus obtained compared to the simulated theoretical values of 798 s^−1^ mM^−1^ [37], where a decreased r_2_* was measured for the 90 nm SAF-NPs compared to this theoretical value.

#### 3.2.2. Computed Tomography (CT)

**Figure 3:**
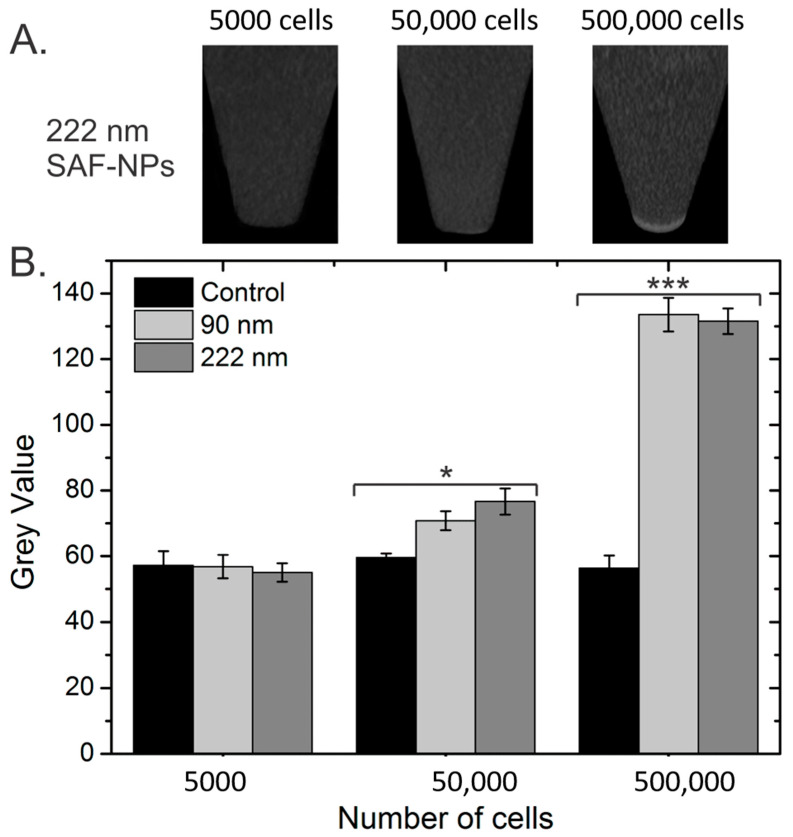
In vitro CT images of the SAF-NP labeled SKOV 3 cells in an agar phantom. (**A**) CT scans of the pelleted SAF-NP-labeled SKOV3 cells for the given cell number and SAF-NP size. (**B**) Grey value for the control (unlabeled), 90 nm SAF-NP and 222 nm SAF-NP samples as a function of the number of cells. * *p <* 0.05, *** *p* < 0.001.

To determine the CT contrast enhancement of the SAF-NP-labeled cells, CT images were taken from the cell pellets in a phantom. At a concentration of 5000 cells, no contrast enhancement was observed between the cell pellet and the agar on top of the pellet (Figure 3). Increasing the number of cells to 50,000, an increase in the gray value was noticed, while for 500,000 cells, a bright pellet was visible. This was quantitatively confirmed by plotting the gray values as a function of the cell number (Figure 3B). Significant contrast enhancement was measured compared to the control cells for 50,000 and 500,000 cells. No distinction could be made between cells labeled with 90 nm and 222 nm SAF-NPs, although the gold concentration per cell was higher for the 222 nm SAF-NP-labeled cells (24.7 pg/cell) than for the 90 nm SAF-NP-labeled cells (18.6 pg/cell).

### 3.3. In Vivo Imaging

To validate the potential of SAF-NPs for in vivo imaging applications, we have used two different experimental models that are of relevance for biomedical applications of contrast agents: (1) intravenous (i.v.) injection of particles; and (2) i.v. injection of pre-labeled cells. For both applications, only 222 nm SAF-NPs were used due to their better T_2_* MRI contrast enhancement.

#### 3.3.1. Intravenous Injection of SAF-NPs

**Figure 4:**
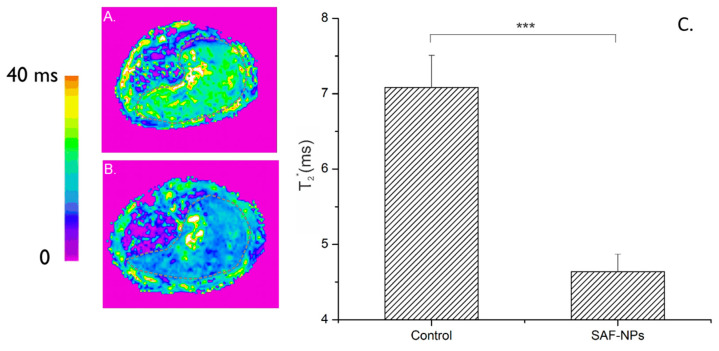
Color-coded T2* maps (MR images) of the liver of a control mouse (**A**) and after SAF-NP injection (**B**). (**C**) The dotted grey line indicates the ROI of the liver. A clear contrast enhancement could be observed after SAF-NP injection, as shown by the significant T2* signal decrease of the SAF-NP-injected liver compared to the control. *** *p* < 0.001.

Parametric T_2_* MRI maps of the liver region, 24 h after injection of 222 nm SAF-NPs into the blood stream (Approach 1), are shown in Figure 4. A clear contrast enhancement in the MRI of the liver is observed for the SAF-NP-injected mice (B) compared to the control mice (A). The T_2_* values decreased from 7.1 ± 0.4 ms to 4.6 ± 0.2 ms (*p* < 0.0001), thus confirming the performance of SAF-NPs as excellent MRI contrast agents in vivo.

**Figure 5:**
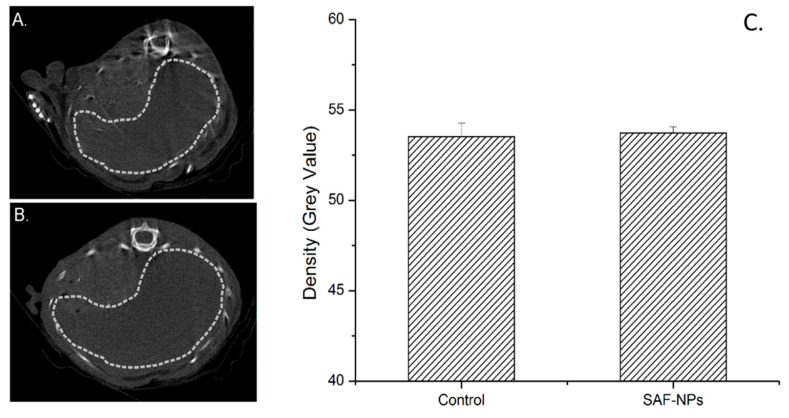
Transversal liver CT images of a control mouse (**A**) and after intravenous SAF-NP injection (**B**). (**C**) The dotted grey line indicates the region of interest (ROI) of the liver. No significant contrast enhancement could be observed after SAF-NP injection, as shown by the significant density of the SAF-NP-injected liver compared to the control.

Using CT, however, no contrast enhancement could be observed in the liver (signal intensities for the liver of control animals: 54.7 ± 0.8; and for the liver of animals receiving SAF-NPs: 54.4 ± 1.2; Figure 5). The presence of SAF-NPs inside the liver 24 h after i.v. injection led to an increased MRI contrast, confirming the high sensitivity of MRI for SAF-NP-induced contrast that was observed in vitro. CT scans of the same animals did not show contrast enhancement in the liver region.

**Figure 6:**
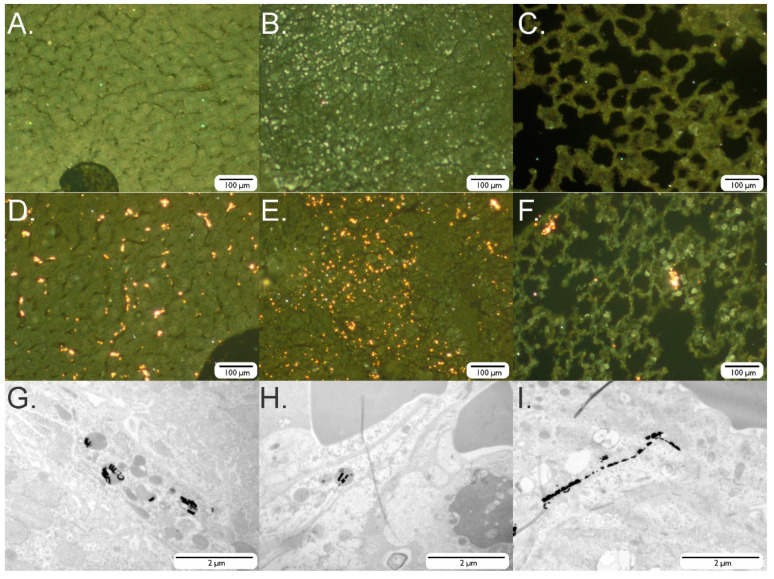
Ex vivo dark-field and TEM images of the liver, spleen and lung cells after injection of SAF-NPs and SAF-NP-labeled cells. (**A**–**C**) Dark-field images of slices of the liver (**A**), spleen (**B**) and lungs (**C**) of control mice. (**D**,**E**) Dark-field and (**G**,**H**) TEM images of a slice of liver (**D**,**G**) and spleen (**E**,**H**) after intravenous injection of SAF-NPs. (**F**) Dark-field and (**I**) TEM images of a lung slice after intravenous SAF-NP labeled cell injection.

Ex vivo dark-field imaging of the liver and spleen confirmed the presence of SAF-NPs whereas the heart (control) and kidney tissue did not show any particle retention (Figure 6).

#### 3.3.2. Intravenous Injection of SAF-NP Labeled Cells

**Figure 7:**
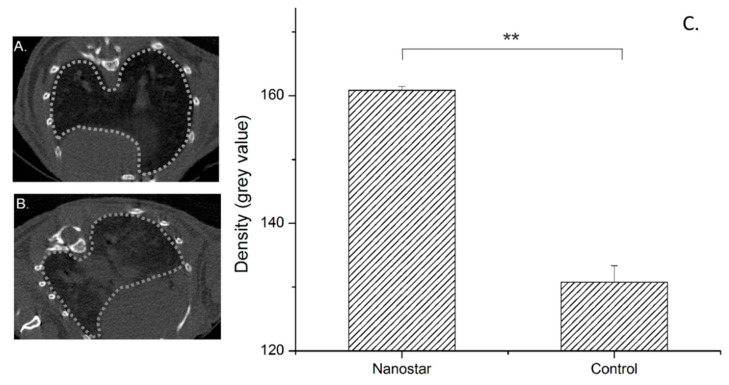
Transverse CT images of mouse lungs after intravenous injection of 500,000 unlabeled (**A**) and SAF-NP-labeled (**B**) SKOV3 cells. (**C**) The dotted grey lines indicate the ROI of the lungs. A positive contrast enhancement was observed after injection of the SAF-NP-labeled cells, as shown in the graph where the density is plotted for both cases. ** *p* < 0.01.

To further explore the potential of SAF-NPs as contrast agents in organs with low background signal in CT images (for example lungs), the SAF-NP-labeled cells were injected intravenously, which should lead to cell accumulation in the pulmonary capillaries of the lungs of the mice [40]. To determine the imaging capabilities of the SAF-NP-labeled cells inside the lungs, CT and MRI scans were acquired 24 h after the injection of the SAF-NP-labeled cells. In Figure 7, transverse CT images of the lungs of a mouse after injection with 500,000 unlabeled (B) and SAF-NP-labeled (A) SKOV3 cells are shown. Contrast enhancement generated by the injection of SAF-NP-labeled SKOV3 cells compared to the unlabeled cells is clearly visible and quantifiable (44.3 ± 2.5 a.u. to 50.8 ± 2.3 a.u. (*p* < 0.01)).

**Figure 8:**
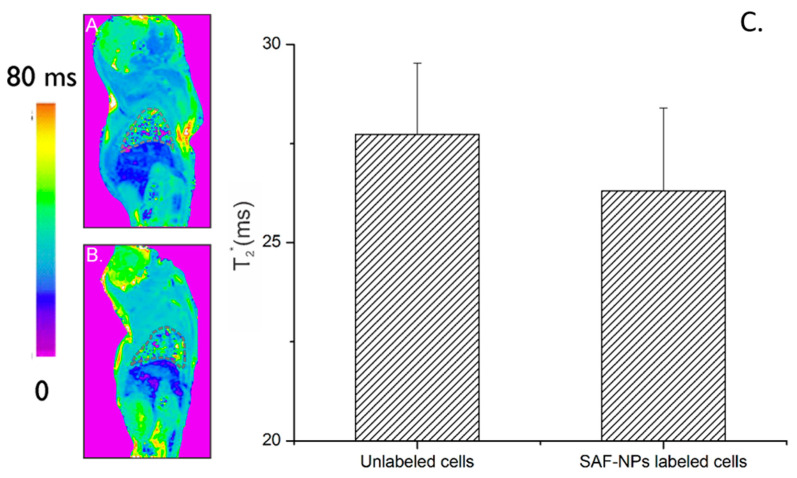
Parametric T2* maps (MRI) of mouse lungs after intravenous injection of 500,000 unlabeled (**A**) and SAF-NP-labeled (**B**) SKOV3 cells. (**C**) The dotted grey lines indicate the ROI of the lungs. No contrast was observed after injection of the SAF-NP-labeled cells, as shown in the graph where the T2* signal is plotted for both cases.

Using MRI, no significant contrast enhancement could be observed in lung tissue of the same animal (Figure 8). For the unlabeled cells, a T2 relaxation time of 27.2 ± 2.4 ms was measured while for the SAF-NP-labeled cells this was 26 ± 3.1 ms. In MRI, the lungs generate a hypo-intense background due to the of protons (for example, tissue water). Therefore, the SAF-NPs inside the SKOV3 cells could not induce an extra signal void (hyperintense contrast enhancement) in the T_2_/T_2_*-weighted MR imaging.

**Figure 9:**
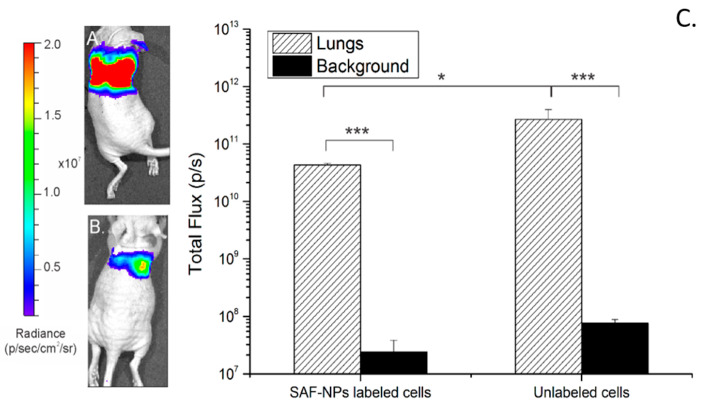
BLI of a mouse after injection of 500,000 unlabeled (**A**) and SAF-NP-labeled SKOV3 cells (**B**). (**C**) A high BLI signal intensity is observed in the lungs of the mouse in both cases, indicating cell accumulation in the lung region. Quantification of the total flux plot on a log scale shows a difference in BLI signal for the mice injected with either 500,000 unlabeled or SAF-NP-labeled SKOV3 cells. * *p* < 0.05 *** *p* < 0.001.

To confirm the distribution of the labeled cells, bioluminescence (BLI) data were acquired 24 h after intravenous injection of 222nm SAF-NP-labeled and unlabeled SKOV3 cells. Figure 9 shows the BLI signal of a representative mouse, 24 h after injection of labeled and unlabeled SKOV3 cells. For the mice injected with unlabeled cells (Figure 9A), a high BLI signal in the lungs is measured (2.67 ± 0.01 10^11^ p/s) compared to the background signal (7.76 ± 1.21 10^7^ p/s). For the SAF-NP-labeled SKOV3 cells (Figure 4B), a smaller (*p* < 0.5; 4.34 ± 0.29 10^10^ p/s) but still significant BLI signal could be observed in the lungs compared to the background (2.40 ± 1.44 10^7^). Of note, a higher BLI signal was observed for unlabeled cells compared to SAF-NP-labeled cells, suggesting that the BLI signal was potentially reduced due to the presence of the SAF-NPs.

The presence of SAF-NPs in the lungs was also confirmed through ex vivo imaging of the lungs, endorsing the BLI results. Hereto, dark-field microscopy and TEM images were acquired from excised tissue ex vivo. After intravenous injection of SAF-NPs, the presence of particles could be confirmed in the liver and the spleen (Figure 6D,E,G,H) when compared to the control samples (Figure 6A,B). Particles also accumulated in the lung tissue (Figure 6F,I), although to a lesser extent when compared with the liver and spleen.

## 4. Discussion

We have demonstrated that SAF-NPs are suitable for in vivo bimodal imaging using CT and MRI. In a cell culture, we were able to show that both the 90 nm and 222 nm in diameter SAF-NPs are readily taken up. Interestingly, the total amount of internalized nanomaterial was comparable for both particle sizes, resulting in only marginal differences in the Au and Ni_80_Fe_20_ amount/cell. This is to some extent unexpected, given the finding that spherical PEGylated particles show optimal uptake conditions at a size of 20–50 nm, and a decreasing uptake in function with an increasing size [41]. For larger particles, it takes longer for the cell to engulf the particle because of slower receptor diffusion kinetics, hence resulting in decreased uptake [42]. However, our SAF-NPs are not spherical and previous studies already have shown that the effect of shape and aspect ratio can lead to different uptake behavior [43]. The disk-shaped SAF-NPs discussed here differ in diameter but consist of the same layered structure and hence have identical thicknesses. One of the possible reasons for similar cell internalization could be that the thickness is the predominant factor that determines the cell uptake efficiency.

Based on our previous work, similar MRI r_2_*-relaxivities were expected for the 90 and 222 nm SAF-NPs [37,39]. However, the larger particles resulted in stronger T_2_* MRI contrast. This difference could be due to the gold capping layers. As the thickness of the gold-capping layer is identical for both particle sizes (10 nm), their effect on the r_2_* reduction will be more pronounced for smaller particles since the proton access to the particles’ stray fields will be restricted [44]. This was also confirmed by the ICP-OES results, as the ratio between the Ni_80_Fe_20_ and the Au content (mass (Au)/mass (Ni_80_Fe_20_) is equal to 4.4 for the 90 nm SAF-NPs and 3.3 for the 222 nm SAF-NPs. For the same particle saturation magnetization (M_s_), the 90 nm SAF-NPs will thus have a smaller magnetic volume fraction, resulting in lower r_2_* values. Nevertheless, with r_2_* values of up to 712 s^−1^ mM^−1^, the SAF-NPs show far better contrast generation performance than others report for combined gold/magnetic nanoparticles [30,31,34,35]. The in vitro SAF-NP performance even outperforms that of the formerly commercially available bottom–up synthetized superparamagnetic contrast agents such as Endorem, with r_2_* values of 655 s^−1^ mM^−1^ [39]. In top–down synthesis, as for the SAF-NPs, the NPs are ‘etched’ out crystal planes. In contrast, bottom–up NPs are synthetized in a nucleation process by adding layers to a substrate. In addition to an easier synthesis, the relaxivity values of the SAF-NPs confirm the increased performance of the top–down synthetized SAF-NPs, inducing a higher magnetic moment because of their larger size compared to bottom–up synthetized nanoparticles. In future research, the top–down approach can be further optimized (e.g., particle shape, material choice, layer thickness) to gain an additional increase in MRI contrast generation. Moreover, the good layer thickness definition of the top-down approach results in a well-controlled 10 nm gold capping layer that can be used for CT contrast enhancement but does not decrease the r_2_* values significantly as is the case for certain bottom-up approaches [36].

Although, the gold concentration in cells internalizing the 222 nm SAF-NPs was slightly higher than for cells internalizing the 90 nm SAF-NPs, no difference in CT contrast was noticed. As explained before, the Au/Ni_80_Fe_20_ ratio is higher for 90 nm SAF-NPs, resulting in a thicker gold layer relative to the particle diameter. As thicker gold layers in hybrid gold/magnetic nanoparticles result in an increased signal attenuation in CT [36], this effect most likely compensates for the lower total gold concentration, resulting in similar performances of 90 nm and 222 nm SAF-NPs in CT.

Comparing the in vitro performance of cells labeled with SAF-NPs for contrast generation in MRI and CT, it is clear that the sensitivity is significantly higher for MRI in our setup. Using MRI, 5000 homogeneously labeled cells in a volume of 200 µL could still be detected, while for CT 50,000 pelleted cells were needed to induce significant signal attenuation. Due to CT’s relative insensitivity to contrast agents compared to MRI, high-contrast loads are required to increase the sensitivity [45]. Thus, a better contrast generation in MRI is expected for the SAF-NPs. However, it must be noted that in vivo imaging is different from imaging phantoms containing cell pellets. Parameters that affect the in vivo MRI contrast are influenced by the microenvironment of the protons and particles, including the cell water content, relaxation times of the surrounding tissue and surrounding macromolecules. As a consequence, contrast generation would be different under conditions mimicking air–tissue interfaces where CT shows a higher sensitivity compared to MRI.

Among the numerous potential in vivo applications of nanomaterials, delivery of therapeutic compounds and monitoring of cells are most frequently suggested for clinical and preclinical use [1,2,3,4,23]. For both applications, knowledge of the temporo-spatial distribution of the nanoparticles is crucial [3,14,23]. Among the various approaches taken for in vivo imaging, superparamagnetic nanoparticles have been used extensively due to low toxicity, high sensitivity and their potential as MRI contrast agents [3,6,23,46]. However, their hypointense contrast is often difficult to visualize, interpret and quantify throughout the body, especially in regions with hypointense background signals, such as the lungs or blood vessels [47,48]. Therefore, we have chosen to combine MRI contrast with an imaging method and contrast agent that would bridge this gap and also provide excellent contrast in the lungs, such as CT imaging [25,26]. For in vivo validation, we have compared two different models: (1) intra-venous (i.v.) injection of SAF-NPs; and (2) i.v. injection of SAF-NP-labelled SKOV 3 cells.

In vivo experiments after i.v. injecton of SAF-NPs confirmed our in vitro findings that showed far better sensitivities in MRI compared to CT, visualizing clearance of NPs via the spleen and liver by using MRI but not by CT. To overcome this lack of sensitivity in CT, higher particle concentrations or a thicker gold capping layer would be necessary. Ex vivo dark-field imaging confirmed the presence of SAF-NPs in the liver and spleen but not in the heart (control) and kidney tissue, proving SAF-NP uptake by Kupffer cells [49] and clearance via the reticuloendothelial system (RES) [50,51]. Our experiments were performed 24 h after intravenous injection after which full RES clearance should be present.

In contrast to i.v. injection of the SAF-NPs, SKOV3 cells labeled with SAF-NPs mainly ended up in the lungs, as demonstrated by CT. No differences in the MR images of the lung were seen due to the hypointense background (see Figure 8), clearly indicating that combining MRI with CT overcomes this limitation of using MRI as a single imaging method. Only small signal changes were observed in the liver by MRI. This is expected, as previous reports indicate that cells are most likely being trapped in lung capillaries [19,52]. These results were confirmed by BLI, which indicated the presences of viable cells in the lungs 24 h after i.v. injection of SAF-NP-labelled SKOV3 cells. Interestingly, the amount of contrast uptake in the liver—which indicates the transfer of SAF-NPs to macrophages that phagocytized dead or dying cells or free NPs due to their release from dying cells [52,53,54]—was lower than in a previous report on i.v. administration of mesenchymal stem cells (MSC) [19]. This indicates better survival of SKOV3 cells than MSC in the mouse lung.

Using SAF-NPs as the cell labeling agent is of interest in the field of stem cell, immune cell and tumour research, where knowledge of the distribution of engrafted cells is of interest. Combining MRI and CT would not only provide information with a higher resolution than the commonly used BLI but has also the potential to be used for larger laboratory animals or in the clinic due to unlimited depth penetration. We thus illustrate the bimodal capabilities of SAF-NPs, of which the magnetic properties can be used to induce MRI contrast enhancement in soft tissues and the gold capping layers can be exploited to enhance CT contrast in areas where MRI has difficulties, such as the lungs. Hereby, one type of nanoparticle can be used for complementary imaging techniques, thereby reducing the invasiveness and difficulties that are encountered using two types of contrast agents in standard MR and CT imaging. Although nanoparticle targeting has limitations in terms of delivery to a target tissue [55], further functionalization of the PEGylated SAF-NPs with peptides, nanobodies or antibodies is feasible, as has been demonstrated for different types of nanomaterials [56,57,58].

## 5. Conclusions

We have illustrated the high potential of bimodal imaging using SAF-NPs for in vitro and in vivo MR and CT imaging. Previously reported experimental SAF-NPs of different sizes showed high uptake by SKOV3 cells, as confirmed by TEM and ICP-OES. With this efficient labeling, 5000 labeled cells could be detected with MRI, resulting in an r_2_* value up to 712 s^−1^ mM^−1^ using the 222 nm SAF-NPs, which outperforms any other reported bimodal gold/magnetic nanoparticles. Moreover, due to the bimodal capacities, significant contrast enhancement in CT was observed, with a detection limit of 50,000 SAF-NP cells under soft-tissue phantom conditions. These bimodal properties were exploited in vivo by two different strategies. Intravenously injected SAF-NPs accumulated in the liver and could be quantified using T_2_*-weighted MR imaging, rendering the SAF-NPs as suitable agents for potential use in hepatic or other soft-tissue imaging. In the lungs, SKOV3 cells labeled with SAF-NPs could clearly be detected using CT imaging, which is the first proof of gold-based CT contrast enhancement in the lungs. SAF-NPs are of particular interest for applications where target sites can be different organs with intrinsic properties that would make imaging by a single method difficult. Such applications are stem or immune cell tracking, but also utilization of functionalized SAF-NPs as tumor-targeting contrast agents. In this work, we unambiguously showed the bimodal imaging capabilities of gold/magnetic SAF-NPs in vivo. SAF-NPs have the potential to become tools in combined imaging approaches where their outstanding performance in MRI can be used to image soft tissue and their CT contrast enhancement properties can be used in organs where MRI is of limited use (lungs, calcified tissue).

## Data Availability

Data supporting reported results will be made available on request.
